# The Role of Polymeric Biomaterials in the Treatment of Articular Osteoarthritis

**DOI:** 10.3390/pharmaceutics14081644

**Published:** 2022-08-06

**Authors:** Carmen Velasco-Salgado, Gloria María Pontes-Quero, Luis García-Fernández, María Rosa Aguilar, Kyra de Wit, Blanca Vázquez-Lasa, Luis Rojo, Cristina Abradelo

**Affiliations:** 1 Departamento de Química y Bioquímica, Facultad de Farmacia, Universidad San Pablo-CEU, CEU Universities, Urbanización Montepríncipe, 28925 Alcorcon, Spain; 2 Instituto de Ciencia y Tecnología de Polímeros (ICTP), CSIC, Calle Juan de la Cierva, 3, 28006 Madrid, Spain; 3 Centro de Investigación Biomédica en Red de Bioingienería, Biomateriales y Biotecnología CIBER-BBN, Instituto de Salud Carlos III, Calle Monforte de Lemos S/N, 28029 Madrid, Spain

**Keywords:** osteoarthritis, polymeric biomaterials, nanoparticles, viscosupplementation, hydrogels, cartilage regeneration

## Abstract

Osteoarthritis is a high-prevalence joint disease characterized by the degradation of cartilage, subchondral bone thickening, and synovitis. Due to the inability of cartilage to self-repair, regenerative medicine strategies have become highly relevant in the management of osteoarthritis. Despite the great advances in medical and pharmaceutical sciences, current therapies stay unfulfilled, due to the inability of cartilage to repair itself. Additionally, the multifactorial etiology of the disease, including endogenous genetic dysfunctions and exogenous factors in many cases, also limits the formation of new cartilage extracellular matrix or impairs the regular recruiting of chondroprogenitor cells. Hence, current strategies for osteoarthritis management involve not only analgesics, anti-inflammatory drugs, and/or viscosupplementation but also polymeric biomaterials that are able to drive native cells to heal and repair the damaged cartilage. This review updates the most relevant research on osteoarthritis management that employs polymeric biomaterials capable of restoring the viscoelastic properties of cartilage, reducing the symptomatology, and favoring adequate cartilage regeneration properties.

## 1. Introduction

Articular cartilage is a flexible connective tissue that is preserved by the synthesis and remodeling of its native extracellular matrix (ECM) [1]. An imbalance between these factors can result in alterations to cartilage composition as well as progressive tissue loss, potentially leading to musculoskeletal disorders, such as arthrosis, rheumatoid arthritis (RA), or osteoarthritis (OA) [2]. An estimated 25% of adults suffer from OA, making it the most prevalent degenerative joint disease. OA is characterized by the progressive degradation of cartilage, subchondral bone thickening, and synovitis, adversely affecting the functional integrity of the joint [3,4]. Subsequentially, 80% of OA patients suffer from limitations to body locomotion, while 25% cannot perform their daily activities, making it one of the ten most disabling diseases in developed countries [3,5]. Its high prevalence due to population aging, as well as its high economic burden, have highlighted regenerative-based therapies for OA as one of the current challenges to be addressed by modern pharmaceutical and medical sciences [6].

OA is caused by an interplay of biomechanical and biological factors (e.g., obesity, trauma, inflammation, or metabolic derangements) in the cartilage matrix, subchondral bone, and synovium. This mechanical stress is detected by articular-related cells, such as chondrocytes and synoviocytes, which, in turn, will respond by releasing inflammatory mediators (e.g., interleukine-1 beta (IL-1β) and tumor necrosis factor-alpha (TNF-α)) and oxidative stress products (e.g., reactive oxygen species (ROS) and nitric oxide (NO). This behavior, combined with an imbalance in cartilage synthesis and remodeling, leads to the degradation and loss of the cartilaginous matrix’s structural glycosaminoglycans (GAGs) and their related proteoglycans [3,7,8]. Due to the complex and poorly understood pathophysiology of OA, conventional treatments are mainly based on symptom prevention. Moreover, drug-based therapies targeting cartilage metabolism and/or subchondral bone remodeling can be used to reduce pain and limit inflammation. Analgesics (e.g., tramadol and paracetamol), corticosteroids (e.g., triamcinolone and prednisolone), and non-steroidal anti-inflammatory drugs (NSAIDs) (e.g., ibuprofen and diclofenac) are considered first-line therapies. However, their use is restricted, due to their unstable nature and poor water solubility. As a result, excessive amounts are needed, potentially leading to systemic side effects, such as ulcers and digestive bleeding [7]. 

To overcome these challenges, novel pharmacological therapies are being created, including disease-modifying OA drugs (DMOADs) (e.g., IL-1β inhibitors). DMOADs are a class of drugs that target the primary pathways in OA, with the intention of delaying or inhibiting disease progression [9]. To be more concise, DMOADs can be classified into three groups: (i) growth factors, (ii) drugs targeting subchondral bone remodeling, and (iii) inhibitors targeting enzymatic degradation and inflammation [10]. With most DMOADs being proteins or protein-derived peptides, polymeric biomaterials are used to obtain a more controlled and/or sustained drug release. This, together with the tertiary structure of proteins and their weak non-covalent bonding, often results in slow kinetics and enhances the low stability in vivo [10,11].

With disease progression, pharmacological treatment may be replaced and/or reinforced with viscosupplementation through the administration of exogenous intra-articular hyaluronic acid (HA) injections. Generally, the synovial fluid (SF) of OA patients contains a lower concentration and molecular weight of HA, compared to a healthy joint. With viscosupplementation, the viscoelastic properties of the synovial fluid can be restored, which, in turn, provides pain relief and makes it possible to reduce the analgesic intake [12,13,14]. In addition, viscosupplementation therapies can benefit from tissue engineering strategies combining biodegradable and highly porous three-dimensional (3D) scaffolds, along with colloidal networks for cell growth and the flow transport of nutrients and metabolic waste [15,16,17]. The research activity in this field is extensive, but only a few developments are being considered for clinical trials [11]. In this review, we report the main contribution of polymeric biomaterial science in the emerging management of OA, including the improvement of (i) viscosupplementation (mainly based on HA), (ii) pharmacological treatments, based on drug delivery systems (comprising micro- and nanoparticles for drug local delivery), and (iii) cell therapy (driven by 3D biomimetic extracellular matrices). For that purpose, we examined a list of selected articles that were collected from a deep search conducted in several scientific databases, such as PubMed (NCBI, US National Library of Medicine), Science Direct© (Elsevier), Web of Science™, and SCOPUS^®^, using different keyword combinations such as “biomaterials”, “polymers”, “osteoarthritis” or “cartilage”, along with additional terms indicative of the system type. Only articles from the last nine years that focused on articular osteoarthritis applications were reviewed.

## 2. Polymeric Systems for Pharmacological Treatments

### 2.1. Local Drug Delivery System Approaches

The reduction in OA symptomatology using drugs, such as analgesic or anti-inflammatory drugs, has traditionally been the first-line treatment of OA [12,18]. Most of these drugs are administered orally, despite their low bioavailability limiting their clinical efficacy and the side effects that this administration route presents [7,19]. On the contrary, the intra-articular administration of injectable systems allows the active ingredients to be locally delivered so that their effect remains in the desired place, with minimized toxicity [19,20]. Therefore, advanced delivery systems that can increase the retention time and maintain sustainable release have been explored, including hydrogels [21,22,23,24,25,26,27,28,29,30,31,32,33], nano- [34,35,36,37,38,39,40,41,42,43,44] and micro-particles [45,46,47,48,49,50,51,52,53,54,55,56,57], combined systems [58,59,60,61], bioactive surfaces [62,63,64] and polymeric drug conjugates [65,66]. Table 1 summarizes the latest developments in drug delivery systems for the encapsulation of anti-inflammatory drugs, antioxidants, and regenerative pro-drugs targeting OA.

### 2.2. Anti-Inflammatory Drugs and Analgesics

Anti-inflammatory drugs and analgesics play an essential role in OA management, as they contribute to the maintenance of a patient’s quality of life [18]. However, each route of administration comes with its own challenges. For example, cellular uptake is highly dependent on the particle shape, size, and surface charge [67], whereas protein drug delivery is often restricted due to its short half-life within the body, making it necessary to use the excessive amounts necessary [68]. To ensure a controlled release with enhanced drug activity, a wide range of drug delivery systems have been developed using a variety of materials, including polymers. 

Poloxamers are amphiphilic synthetic polymers that form micelles. In the work of Dos Santos et al. [27], a poloxamer established the electrostatic interactions between its hydrated micellar corona and tramadol to module the drug release kinetics. The combination of different poloxamer PL140/PL188 ratios resulted in a series of hydrogels that were intended for the delivery of tramadol. Similarly, tramadol delivery could be extended by using a thermoresponsive chitosan/poloxamer/glycerophosphate hydrogel comprising inner nanocavities for drug retention [26]. Steroidal anti-inflammatory drugs, such as bupivacaine and dexamethasone, were also encapsulated into HA-PL407 and HA- Poly(ethylene glycol dimethacrylate) (PEGDMA) hydrogels, respectively, with controlled delivery kinetics due to HA-polymer interactions and the precise control of HA concentration and molecular weight. In another way, triamcinolone-controlled release in response to SF enzymes (e.g., metalloproteinases) was achieved using the amphiphilic molecules of triglycerol monostearate (TG-18), which were able to self-assemble, forming an arthritis flare-responsive hydrogel that releases different drug concentrations, depending on its exposure to metalloproteinases. In this way, when the enzymatic activity reaches non-pathological levels, hydrogel degradation slows down and delays triamcinolone release, so as to prolong its residence time and effects [23].

In addition to hydrogels, micro- and nanoparticles offer a drug delivery system that is widely considered for the management of OA. Nanotechnology has been shown to extend a drug’s residence time, allowing targeted delivery, as well as increasing physiochemical stability [7]. That being said, several authors have used nanoparticles to enhance the anti-inflammatory effects of drugs. To increase aceclofenac bioavailability in the knee joint, solid lipid nanoparticles (SLNs) were conjugated with chondroitin sulfate (CS), a ligand that establishes specific interactions with CD44 receptors, annexin, and leptin receptors [36]. Likewise, Crivelli et al. [37] prepared silk fibroin nanoparticles (SFNs) loaded with two celecoxib concentrations (5% and 11% *w*/*w*), showing that celecoxib was released over a longer period when the SFNs were loaded with a higher drug concentration. Hence, a time-dependent release system was obtained, wherein an increase in drug loading led to a longer release time, rather than a higher dose release. In another work, El-Gogary et al. developed celecoxib-loaded hyaluronan nanoparticles using the nanoprecipitation method, optimizing the nanoparticles in terms of size and encapsulation efficiency, and demonstrating superior in vivo performance regarding the histological, swelling, and immunohistochemical parameters in an OA rat model [40].

The critical role of oxidative stress products such as ROS in the development and progression of OA has led to the development of different ROS-responsive systems. Zhang et al. [41] established a system that consists of ROS-responsive polythioketal urethane nanoparticles, incorporating dexamethasone in the core, which were capable of scavenging several kinds of ROS, along with triggering the degradation of the polymer. Dexamethasone has also been loaded into poly(lactic-co-glycolic acid) (PLGA) nanoparticles using the nanoprecipitation method, showing sustained drug release over ten days, as well as better in vivo anti-inflammatory properties compared to free dexamethasone [69]. Besides, dexamethasone, celecoxib, and tenoxicam have also been encapsulated into terpolymer nanoparticles, based on a methacrylic derivative of vitamin E, vinylpyrrolidone, and vinyl caprolactam [42]. The systems were optimized in terms of their hydrodynamic properties, encapsulation efficiency, and in vitro biological performance, as well as in terms of anti-inflammatory effect by the reduction of inflammatory factors. In this case, the anti-inflammatory properties of the encapsulated drugs were maintained after the encapsulation process and, at the same time, a reduction of the free-drug toxicity was achieved, both in chondrocytes and macrophages. Similarly, different types of microparticles, mainly microspheres, were used to tune the drug delivery to extend its therapeutic effect. To prolong the drug effect, lornoxicam-loaded chitosan-tripolyphosphate (TPP) microspheres were prepared via ionotropic gelation, based on the electrostatic interactions set between the amino groups of chitosan and the anionic groups of TPP, a crosslinker agent [45]. Other authors synthetized PLGA or polycaprolactone (PCL) microspheres with various polymer/drug ratios (1:1, 2:1, and 3:1) and concluded that an increase in polymer concentration led to the formation of less porous networks, through which aceclofenac was slowly released [46]. Finally, polyester amide (PEA) microspheres were able to control the release of the drug through a self-regulated process in which PEA degradation slowed down when the inflammation decreased. Thus, a controlled release of the active ingredient was achieved in response to tissue inflammation [49,50]. Han et al. prepared diclofenac-loaded injectable hydrogel microspheres, synthesized by dip-coating a self-adhesive polymer on a superficial surface of photo-crosslinked methacrylate gelatin hydrogel [57]. The gel could protect articular cartilage and alleviate inflammation, due to the system lubrication and sustained drug release.

The combination of hydrogels and polymeric nanoparticles can result in a binary release system that extends drug release by a diffusion process while, on the other hand, allowing the encapsulation of various active ingredients and their subsequent release at different times and/or rates. To verify the aforementioned information, several particle/gel systems were developed. The combination of chitosan hydrogels with alginate microspheres has revealed that the increase of the microsphere concentration, resulted in the control of hydrogel viscosity and, thereby, diclofenac release could be tuned [61]. Separately, melatonin-modified chitosan microparticles were loaded with prednisolone and dispersed in alginate/carboxymethyl cellulose (CMC) hydrogels [60]. This system not only allowed researchers to delay both melatonin and the active ingredient release but also to extend their effect over time. However, melatonin was more exposed to degradation than methylprednisolone because of its direct conjugation to chitosan and, thus, was released earlier. Lastly, chitosan hydrogels containing lipid nanocapsules (LNCs) [59], and PEG/PLGA hydrogels loaded with PLGA microspheres were able to sequentially release two active ingredients, locating one of them in the matrix of the hydrogel and the other in the core of the particles. Furthermore, in the second system [58], matrix-located dexamethasone was first released and then caused the vasoconstriction of the injection site, to improve bupivacaine bioavailability by preventing its diffusion to other tissues. MbS2 nanosheets were essential to building chitosan-modified molybdenum photo-responsive carriers that enabled scientists to control dexamethasone release by adjusting the NIR radiation [62]; in the second system, different cyclodextrin polymers (α- CD, β-CD y γ-CD) were used to produce liquid or solid (implantable discs) systems that were capable of establishing specific drug-cyclodextrin interactions to extend the release of several active ingredients, such as hydrocortisone, triamcinolone or dexamethasone [64]. 

### 2.3. Antioxidants

The chronic inflammatory condition that accompanies OA progression leads to the production of pro-inflammatory cytokines, such as interleukin IL-1β. This is the most active cytokine during disease progression and stimulates the production of ROS (e.g., hydroxyl, peroxides, and superoxides) and NO [8]. As a further matter, oxidative stress triggers lipid peroxidation and causes damage to chondrocyte DNA, inducing a homeostatic imbalance that promotes the degradation of the main structural component of cartilage, type II collagen [8,70]. Therefore, decreasing ROS levels with the right antioxidant could delay the progression of OA. In general, the materials designed to improve antioxidant delivery were the same as those for anti-inflammatories. 

Several hydrogels, with different properties and compositions, were developed to deliver antioxidants. The study of the behavior of L-polyalanine (PA)/methoxy-PEG hybrid polymers established that while PA was packed in β-sheets, methoxy-PEG remained flexible and hydrated [32]. As the temperature increased, methoxy-PEG became stiffer, while the sheets aggregated to form hydrogels capable of sustaining that were quercetin delivery. On the other hand, Songkroh et al. synthetized chitosan hydrogels chemically crosslinked with genipin for curcumin delivery [31]. They added anionic sodium salts, such as sodium bicarbonate or sodium sulfate, to reinforce matrix crosslinking by forming ionic bonds with chitosan to delay curcumin release. Finally, a study developed hydrogels where carboxymethyl hexanoyl chitosan (CHC) micelles interacted with each other due to the incorporation of HA, which acts as an inter-colloidal crosslinking agent [33]. These hydrogels showed the ability to release berberine depending on pH variations that arose with the course of the disease. In the case of OA, the joint pH decreases; thus, the hydrogel matrix was less degraded and berberine release was maintained over time. 

Several authors studied the delivery of antioxidants by using different nanoparticles. Curcumin, one of the most recently studied antioxidant molecules, has been encapsulated in different delivery systems. For example, the previously mentioned terpolymer nanoparticles used by Pontes-Quero et al. to encapsulate celecoxib, dexamethasone and tenoxicam were also used to encapsulate curcumin [43]. These curcumin-loaded amphiphilic systems showed remarkable radical scavenging activities in both nude and loaded nanoparticles and in vitro anti-inflammatory effects through the reduction of NO and other inflammatory factors. Furthermore, Kang et al. developed acid-activable polymeric nanoparticles based on poly (β-amino ester) loaded with curcumin. This system takes advantage of the lower pH present in OA joints to trigger the hydrophobic/hydrophilic transition of tertiary amine groups achieving a controlled release of curcumin under acidic conditions [44]. Silk fibroin nanoparticles, designed to release celecoxib, were also able to internalize curcumin in their cores to extend the antioxidant release over time [37]. Alternatively, chitosan/HA nanoparticles with dense networks were developed, thanks to the ionic crosslinking set between chitosan and a polyphosphate, in order to slow down curcumin release [39].

Since nanoparticles can modify the speed of antioxidant release, several articles discussed the possibility of using microparticles to achieve the same effect. On the one hand, the establishment of non-covalent interactions (e.g., hydrogen bonds) between the amino groups of the polymer and the hydroxyl groups of the active ingredient resulted in a rutin-loaded microsphere system that extended the release of rutin [51]. Likewise, silk fibroin/HA microspheres made with different silk fibroin/HA ratios (78:22, 83:17, and 88:12) [54] and gelatin/silk fibroin microspheres [53] demonstrated that a higher silk fibroin proportion increased the retention of curcumin because of the interactions that the antioxidant established with silk fibroin hydrophobic domains. Moreover, in the case of gelatin, the addition of silk fibroin delayed microsphere degradation, since its hydrophobic β-sheet structures are very stable [53].

### 2.4. Immunosuppressive and Antirheumatic Drugs

OA is characterized by an imbalance in anabolic and catabolic processes maintained by chondrocytes, mainly evoked by increased production of pro-inflammatory cytokines (e.g., IL-1β y TNF-α). Within the joint, the cytokines stimulate the production of matrix metalloproteinases (MMPs) and disintegrin metalloproteinases, playing an essential role in the degradation of structural components in articular cartilage, such as collagen type II, aggrecans, and GAGs [71]. Overall, disease-modifying anti-rheumatic drugs (DMARDs) were reported to have the ability to decrease the expression of these pro-inflammatory cytokines and MMPs, both in vitro and in vivo [34,55,65,66]; thus, inflammatory cytokines are currently being assessed as potential biomarkers in the diagnosis and management of OA. Although their use in the treatment of OA has not yet been approved, immunosuppressors have been considered potential therapeutic approaches for the prevention of cartilage degradation and OA progression [72]. Studies have revealed that rapamycin, a clinically used immunosuppressant for organ transplantation patients, has shown immunosuppressant properties by activating the mechanism of autophagy through the mammalian target of rapamycin (mTOR) inhibition. Autophagy is a process by which a cell degrades dysfunctional intracellular components, such as organelles or proteins. This degradation system is essential in the energy regulation of the cell, making it a mechanism of survival [71,72]. Moreover, De Luna-Preitschopf et al. [71] constructed an in vitro OA model of patient-derived chondrocytes, cultured in a TNF-α or IL-1β supplemented medium, to determine the immunomodulatory properties of mTOR inhibition. The study concluded that rapamycin inhibits the synthesis of inflammatory cytokines, prevents extracellular matrix degradation, showed chondroprotective effects, and prevents chondrocyte death. Similarly, Takayama et al. [72] showed that a local intra-articular rapamycin injection in an in vivo murine model significantly delayed articular cartilage degeneration. This route of administration has the advantage of local administration, with maximized efficacy and minimized toxicity [73,74]. Thus, injectable hydrogels have been studied as a potentially effective and safe drug delivery system including, but not limited to, a gelatin hydrogel containing rapamycin micelles, which was reported to have a longer and more controlled release, compared to a single rapamycin injection in mice [28].

On the other hand, using chitosan and glutaraldehyde together in the same system favored the interaction between their respective amino and aldehyde groups and, subsequently, promoted the formation of dense matrix nanospheres for the extended release of the immunosuppressor methotrexate (MTX) [35]. Likewise, MTX-controlled release can be delayed through lipid nanocapsules (LNCs) [34]. Moreover, the LNCs increased MTX bioavailability and allowed the maintenance of anti-inflammatory effects at doses 75% lower than conventional methotrexate injections. Finally, the development of microspheres from several PLGA polymers made it possible to extend the release of leflunomide by establishing hydrophobic interactions between drug and the polymer [55].

Additionally, other methods were used to deliver DMARDs, mainly in the form of HA conjugates. MTX was grafted to HA through its α-carboxylic acid, using a peptide-ethylene diamine linker (α-Phe-Phe-CH_2_-NH_2_), and was later incubated with the SF of osteoarthritic rabbits [65]. The authors concluded that at acidic pH levels, cathepsins found in the SF were able to cleave the α-bond and cause the release of MTX. Moreover, the study showed that, when MTX was combined with HA, the hematological toxicity of the drug was reduced. Kim et al. focused their research on studying the release profile of sulfasalazine (SASP) from sulfasalazine-containing HA systems [66]. The developed HA conjugate released SASP up to sixty-three days after its administration, suggesting that the electrostatic interactions established between HA negative charges and SASP-positive charges promoted drug retention and extended its release over time. 

## 3. Polymers Used in Viscosupplementation

### 3.1. Natural-Based Polymers

**Figure 1:**
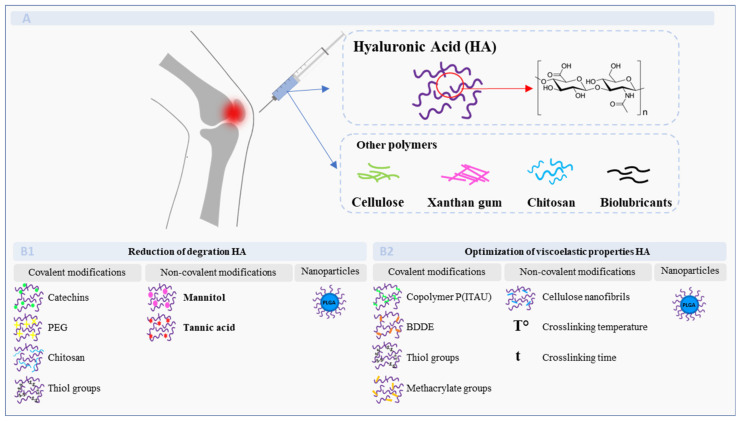
Biomaterials in the development of viscosupplementation strategies targeting OA. (**A**) Recent viscosupplementation strategies include the polymers: hyaluronic acid, cellulose, xanthan gum, chitosan, as well as biolubricants. (**B1**) Hyaluronic acid may be altered by (non-) covalent modifications and/or can be used in nanoparticle strategies to reduce the degradation of HA in vitro and/or in vivo. Covalent modifications include catechins, PEG, chitosan, and thiol groups. Non-covalent modifications are the addition of mannitol or tannic acid. (**B2**) To optimize the viscoelastic properties of HA, (non-) covalent modifications containing copolymer P(ITAU), BDDE, thiol groups, methacrylate groups, cellulose nanofibrils, and PLGA nanoparticles, as well as the crosslinking temperature and time, have been used.

In OA patients, the decrease in concentration and molecular weight of HA leads to the loss of the viscoelastic and lubricant properties of SF, which promotes joint degeneration [12,13,14]. Proper lubrication of the cartilage is essential to maintain its function and alleviate the symptoms of damaged tissue. The components of the SF implicated in the wear protection of cartilage are mainly natural polymers, such as HA, lubricin, and aggrecans [75]. Therefore, a common method to restore the function of the joint is HA injections, also known as viscosupplementation. HA is a favorable material for the treatment of OA due to its viscoelastic properties and biocompatibility [18]. However, non-modified HA presents poor mechanical properties. Thereby, it is necessary to modify its structure through covalent or non-covalent modifications [76,77,78,79,80], in order to improve its degradation rate and extend its residence time. Drug delivery systems incorporating HA present an alternative method to extend functional residence time in vivo, as well as allow a controlled release [11,77,81,82,83,84,85,86]. Other biomaterials that show promising features in the development of viscosupplementation components are based on cellulose hydrogels [17,87,88], xanthan gum [89], biolubricants [90], or chitosan-based interpenetrating polymer networks (IPNs) to restore cartilage mechanical properties (Figure 1) [91,92,93,94,95]. 

### 3.2. Hyaluronic Acid

HA is a natural polysaccharide that helps to maintain the viscoelastic properties of the SF. Correspondingly, it allows the SF to act as a shock absorber when the joint is submitted to high loads and acts as a lubricant during low loads. However, osteoarthritic patients present a reduction in both the concentration and molecular weight of HA, impairing its viscoelastic properties and preventing the synovial fluid from performing its lubricating functions [12]. Therefore, the intra-articular injection of exogenous HA is one of the main strategies to reduce and palliate OA symptoms. However, the efficient lymphatic system of the joints rapidly cleared out HA-based viscosupplementation from the joint cavity due to its rapid degradation, which was caused by the combined effect of hyaluronidases and oxidative stress products [89]. Thus, with the aim of improving the stability and tribological properties of injectable formulations, several covalent and non-covalent modifications of HA have been performed to recover the lubricating and shock-absorbing properties of SF [96]. Some HA modifications are, for instance, grafting poly(itaconic anhydride-co-3,9-divinyl-2,4,8,10-tetraoxaspiro undecane) to HA chains [79], the insertion of thiol groups into HA for disulfide bridge formation between polymer chains, using cysteine ethyl ester [77], or incorporating different equivalents (1 to 4) of methacrylated groups to HA hydroxyls to synthetize photo-crosslinkable hydrogels [76]. In addition, Zheng et al. grafted 2-methacryloyloxyethyl phosphorylcholine, a zwitterionic material with important lubricating properties, onto low and high molecular-weight (LMW and HMW) HA. This system showed enhanced biolubricating and anti-inflammatory properties by the upregulation of cartilage anabolic genes and the downregulation of cartilage catabolic genes [97]. Alternatively, the addition of tannic acid, manitol [85,98], or epigallocatechin (EGCG) monomers [81,83] to HA promoted the formation of non-covalently crosslinked hydrogels with antioxidant capacity, due to their ROS scavenging activity. In all the studied cases, hydrogels were less sensitive to enzymatic degradation, with an increased crosslinking rate. From this, it can be concluded that HA modifications have an impact on the hydrogel degradation rate.

Furthermore, active molecules, such as anti-inflammatory or antioxidant molecules, can be loaded into HA hydrogels to obtain a combined effect of viscoelasticity and the effect of the target active molecule. For instance, Diaz-Rodriguez et al. developed beta-lapachone, an anti-inflammatory and wound-healing promoter molecule, in loaded hydrogels, based on thermosensitive polymers (poloxamers) combined with HA, which showed in an ex vivo OA model the restoration of the rheological properties of synovial fluid, while decreasing inflammation [99]. Furthermore, Gao et al. used camptothecin nanocrystals, an antiproliferative chemotherapeutic agent, loaded into in situ-forming injectable hyaluronic acid hydrogels. The hydrogels demonstrated a permanence in the joint of over four weeks, as well as reduced IL-1β levels [100]. In other work, epigallocatechin-3-gallate, a modulator of inflammation and scavenger of radical species, was combined with tyramine-conjugated HA and gelatin to create a composite hydrogel [101]. The hydrogel provided protection to chondrocytes against IL-1β and led to chondrogenic regeneration in vitro.

### 3.3. Cellulose

Several authors have studied the possibility of using cellulose and its derivatives for viscosupplementation, due to its biocompatibility, non-toxicity, water-holding capacity, and superior mechanical properties [102,103]. For instance, amidated carboxymethylcellulose (CMCA), in the form of free polymer solution (CMCAp), and its crosslinked hydrogel derivative (CMCAg) were blended at different polymer/gel ratios (from 0/10 to 10/0); they achieved similar rheological properties of SF and were suitable lubricant substitutes for articular cartilages [17,87]. Boyer et al. prepared injectable, self-hardening, mechanically reinforced hydrogels composed of silanized hydroxypropyl methylcellulose (Si-HPMC) mixed with silanized chitosan [104]. This hydrogel was able to support, both in vitro and in vivo, the viability and activity of adipose stromal cells as well as the regeneration of osteochondral defects in dogs.

IPNs have been used to improve the restoration of damaged cartilage, by retaining SF into damaged cartilage and increasing ECM stiffness. For example, Suo et al. prepared chitosan and gelatin semi-IPNs that were later transformed into full IPNs by undergoing an alkalization process. The growing polymer concentration resulted in a denser network that improved the viscoelastic properties of the hydrogel. Alternatively, alginate and polyacrylamide IPNs were synthetized with two different concentrations of bis acrylamide (0.03 and 0.06%) as a crosslinking agent, leading to improved viscoelasticity. Nonetheless, the results indicated a higher friction coefficient and greater wear due to reduced lubrication [92]. Furthermore, different concentrations of silica nanoparticles (1, 2 and 4%) were added to the alginate/polyacrylamide IPNs [91] and resulted in a dose-dependent increase in viscoelasticity and wear resistance, which made it possible to rectify the previous formulation limitations. Another example is the semi-synthetic IPN developed by Cooper et al., based on a GAG-inspired zwitterionic polymer, 2-methacryloyloxyethyl phosphorylcholine, and collagen fibrils created through in situ photopolymerization [105]. This IPN was able to improve lubrication by augmenting the biphasic tissue’s interstitial fluid phase, as well as by the friction dissipation of the tissue’s solid matrix.

### 3.4. Other Polysacharide-Based Materials

Next to HA and IPNs, there is a wide range of other materials that are being developed for viscosupplementation. Among them is a gellan gum hydrogel coated with polyvinyl alcohol (PVA) [106], which showed rheological characteristics comparable to commercial formulations and was non-cytotoxic to chondrocytes. Additionally, lactose-modified chitosan has been assessed by multiple authors because of its particular biological and physical-chemical properties. Scognamiglio et al. [107] prepared lactose-modified chitosan hydrogels, reticulated with boric acid, which demonstrated rheological properties similar to the commercialized products. In addition, the hydrogel expressed a higher resistance to chemical degradation when compared to HA systems. Salamanna et al. [108] illustrated that lactose-modified chitosan, combined with HA, can be used to achieve significant improvements in articular cartilage degeneration and synovium inflammation in a rat model. Moreover, the hydrogel demonstrated that it was better able to withstand degradation, compared to non-modified HA, exhibiting both in vitro biocompatibility and antioxidant activities. Furthermore, calcium ions. crosslinked with gellan gum and hyaluronan formulations. loading the analgesic oleuropein were prepared by Consumi et al., which compounds were also demonstrated to have suitable viscosity, antioxidant activity, and cytocompatibility properties [109].

## 4. Cell-Based Therapies for Osteoarthritis 

### 4.1. Polymeric Scaffolds

Due to the lack of vascularization, the dense ECM, and the limited number of chondrocytes, the ability of the cartilage to regenerate may result in a slow and difficult process [70,110]. In addition, with disease progression, the use of viscosupplementation would become insufficient and even inefficient since the absence of ECM production would significantly intensify its degradation. In consequence, several techniques, such as autologous chondrocyte implantation (ACI) or matrix-guided autologous chondrocyte implantation (MACI), are used for chondrocyte implantation in damaged cartilage to allow the production of regular hyaline cartilage. Nevertheless, these techniques require the prior removal of healthy cartilage tissue for subsequent implantation in the affected area [110]. Hence, one of the main advantages of using injectable hydrogels is their non-invasive implantation [111], in addition to the possibility of mimicking a three-dimensional cartilage ECM structure that may allow the maintenance of cell phenotypes and, therefore, may be suitable for the replication and regeneration of the damaged tissue [110,112].

Table 2 summarizes recent studies, based on cell regenerative therapies, that have mainly focused on the influence that the modification of hydrogel properties, or the addition of components, may have on cellular viability, proliferation and differentiation. Some examples are the influence of the modification of the molecular environment on incorporated chondrocytes [113,114,115,116,117,118,119,120,121,122,123,124] or the encapsulation of mesenchymal stem cells (MSCs) [125,126,127]; or the changes produced in the components of the cellular environment [125,128,129,130,131,132] and in the physical properties of gels [133,134,135,136].

### 4.2. Chondrocyte-Loaded Polymeric Scaffolds

Chondrocytes are the main cellular element of cartilage and have the role of maintaining the cartilaginous matrix through the production of type II collagen and GAGs, the main components of cartilage ECM. Therefore, for chondrocytes to proliferate and synthesize ECM components, biomimetic regenerative hydrogels must recreate that environment by adding molecules capable of setting up specific interactions with the cells [111]. Through several hydrogel modifications, several articles evaluated the influence of the cellular microenvironment on chondrocyte behavior. The addition of CS nanoparticles to a hydrogel composed of semi-IPNs of alginate and polyvinyl acetate (PVA) [121], as well as the synthesis of pullulan and CS hydrogels with different CS/pullulan ratios [116], demonstrated that CS promoted the maintenance of the chondrocyte phenotype and the creation of new hyaline cartilage, mainly through ECM production. In the pullulan and CS hydrogel [116], these effects were observed as the amount of CS increased and at the optimum ratio between both components. The influence of CS, by itself or in combination with HA, was also examined in fibrin and alginate hydrogels [117], verifying that both HA and CS are capable of increasing ECM secretion, as well as cell proliferation. Statistical analysis suggested that the effects of both components on cell proliferation could be additive, contrary to their effect on ECM production. In addition, a study of elastin and HA hydrogels, where the mechanical properties remained constant even when HA concentration varied, concluded that an increment in HA concentration resulted in more effective maintenance of the chondrocyte phenotype, an increase in hyaline cartilage production and a tissue degradation decrease. This HA ability is probably due to its interaction with chondrocyte CD44 receptors [122]. On the other hand, the addition of type II collagen to the cell environment improves the maintenance of the chondrocyte phenotype and ECM production, as well as cell proliferation [119]. For this reason, even if a particular article described an increase in cartilage production as the consequence of TGF-β1 addition to type II collagen/HA hydrogels, further studies are required to determine the real influence that this growth factor has on cells, as the observed results might be due to its combined action with type II collagen [115]. Lastly, chondrocyte metabolic activity in HA hydrogels can be increased in a dose-dependent manner, through elastin insertion on the polymer chain, mainly due to elastin adhesion domains that can be recognized by the cells, emulating the ECM microenvironment [113]. On the other hand, chondrocytes encapsulation in type I collagen hydrogels at different concentrations (5, 7, and 10 mg/mL) confirmed that an increment in this type of collagen stimulates chondrocyte transdifferentiation to fibroblasts, as well as chondrocyte hypertrophy, thereby directly affecting the normal production of cartilage [114]. 

Depending on its location, each tissue shows specific mechanical properties that the ECM translates to cells, through a mechanism known as mechanotransduction. These mechanical signals regulate different cellular processes, such as cell proliferation and differentiation and, therefore, the modification of any of them has an impact on cell behavior [111]; thereby, changes in the hydrogel mechanical properties have an influence on chondrocytes. By modifying polymer molecular weight, it is possible to obtain alginate/CS Sr^2+^ crosslinked hydrogels with LMW and HMW [118]. The LMW hydrogel has a porous and less viscoelastic matrix, since alginate establishes fewer ionic bonds with Sr^2+^, due to its lower molecular weight. The viability, metabolic activity and proliferation of chondrocytes is greater in LMW hydrogels because their porosity promotes cartilage formation by improving the exchange of nutrients and gases, as well as metabolic waste elimination. On the other hand, hybrid PEG/polyalanine (PA) hydrogels with different β-sheet secondary structures were synthetized [120]. The increase in the polyalanine chain length encouraged *β*-sheet formation while sheet-packing into fibrillar-like microstructures, due to the hydrophobic interactions formed between the PA methyl groups of different sheets. Thanks to this fibrillar microarchitecture, similar to that of cartilage ECM, the lengthening of the PA chain stimulated chondrocyte cartilage production. However, these effects are not proportional to polymer chain length because, when the chain is too long, ECM deposition decreases, and the expression of chondrocyte transdifferentiation markers increases. 

### 4.3. Mesenchymal Stem Cell-Loaded Scaffolds 

Nowadays, the use of MSCs for the development of new therapies in the field of regenerative medicine is moving further to the forefront of research. Their easy isolation, high proliferative capacity and ability to differentiate into various cell types [111,137] are some of the advantages of MSCs. The effectiveness of MSCs treatments on cartilage regeneration can only be achieved when the chosen scaffolds can control the differentiation of cells into chondrocytes.

Due to their already mentioned characteristics, hydrogels are the most appropriate materials for MSC encapsulation and differentiation. Several studies have verified that hydrogels provide suitable scaffolds for cell differentiation [125,126,127]. They demonstrated that MSCs proliferate and express genetic chondrogenic markers but not hypertrophy markers. One of the articles [126] confirmed the production of ECM by differentiated cells, through the detection of secreted GAGs, whereas another [127] noted a significant reduction in the cartilage degradation markers (i.e., MMPs).

As cell differentiation is a complex process wherein physical and biochemical signals are barely replicable [111], the modification of any of the hydrogel parameters may affect both the scaffold and the cells. The influence that modifications of the biochemical environment have on cell differentiation has been studied by different authors. For instance, by adding fibroblast-derived ECM to an alginate hydrogel containing MSCs, the expression of chondrogenic markers and cartilage production increased [130]. In this case, the adhesion and signaling molecules found in the ECM created a microenvironment that had a direct effect on cell differentiation. On the other hand, PEG/lactic acid hydrogels capable of improving cell differentiation were synthetized by adding TGF-β3 and HA to the mix [129]. Both TGF-β3 and HA play an important role in MSC differentiation into chondrocytes, as TGF-β3 is an essential growth factor and HA interacts with the cellular CD44 receptors, promoting chondrogenesis. Furthermore, the influence of TGF-β1 on chondrogenesis was studied in methacrylated-chitosan hydrogels into which type-II collagen was also incorporated. Subsequently, cellular condensation and the deposition of GAGs increased, due to the presence of type-II collagen, which enhances cell-matrix interactions, and TGF-β1, stimulating chondrogenesis. However, the results achieved with TGF-β [128,129] are the consequence of the combined action of two components, either TGF-β and HA or TGF-β and type-II collagen; therefore, more studies are required to evaluate the effect of each component on chondrogenesis. The effects observed on MSCs encapsulated in methacrylated HA hydrogels containing CS or type I collagen were similar; cell differentiation and chondrogenic marker expression increased, as well as cartilage production, while the expression of hypertrophic and degradation markers was reduced [132]. Finally, chondrogenesis can be modified by adding glucosamine, a precursor of GAG synthesis. The synthesis of PEG hydrogels, supplemented with growing concentrations of glucosamine (2, 5, 10, and 15 mM), proved that glucosamine encouraged MSCs differentiation to chondrocytes and ECM secretion, especially GAG secretion [131]. It should be noted that the effects of glucosamine are not proportional to its concentration, since the best results were observed at medium concentrations. 

Like chondrocytes, the physical and biochemical properties of hydrogel have a major influence on MSCs behavior; therefore, several hydrogel modifications have been studied to tune their characteristics and thereby drive their differentiation pathways and fate. HMW HA hydrogels, crosslinked with divinyl sulfone at different concentrations, revealed that a greater number of crosslinks results in an improvement in the hydrogel elastic properties and the formation of a more compact network. Both are caused by the reduced intrinsic mobility of the polymer chains [133]. However, as divinyl sulfone concentrations increased, cell proliferation slightly decreased, since the hydrogel compact network structure hindered the exchange of oxygen and nutrients. On the other hand, PEG was crosslinked with LMW or HMW poly(lactic acid) (PLA) to create photopolymerizable hydrogels [135]. The results demonstrated that LMW PLA hydrogels had stiffer matrices that negatively affected MSCs proliferation because nutrient and water diffusion through the scaffold was restricted. Despite this issue, the material’s stiffness did not impair cell differentiation since no significant differences were observed in chondrogenic marker expression or ECM production. Similarly, hydrogel stiffness and degradation rates can have a combined effect on MSC chondrogenesis. For example, in a gel containing gelatin, crosslinked with ethyl lysine diisocyanate (LDI), a high LDI concentration led to a higher density environment and slowly degrading hydrogels [134]. In the in vitro studies performed with MSCs, both hydrogels promoted cell chondrogenesis but especially the less dense and rapidly degrading ones. In vivo, cell differentiation is slowed down, causing a fibroblast-like appearance and a decrease in ECM production. However, due to its structural stability, the denser and more slowly degrading hydrogels are the only scaffolds capable of maintaining the mechanical cues that are essential for chondrogenesis. Two types of methacrylated collagen hydrogels with the same composition and similar mechanical strength, but with different network microstructures (one fibrous and the other porous), were prepared by Yang et al. [136]. The fibrous network promoted both in vivo and in vitro chondrogenic differentiation and prevented the osteogenic differentiation of MSCs, as the expression of osteogenic markers decreased. Nevertheless, the fibrous network could not inhibit the expression of cell hypertrophy markers. In the light of the results, it seems that the fibrous network hydrogel imitates the ECM fibrous structure, which is made of collagen and elastin, enhances cell-matrix interactions and promotes chondrogenesis. In consequence, the hydrogel microstructure is considered an essential factor to control cell differentiation; while the fibrous network promoted chondrogenesis, the porous network promoted osteogenesis.

## 5. Conclusions and Final Remarks

The advanced developments reviewed herein for the treatment of articular OA show their capacity to enhance the mechanical stability of synovial fluids, providing improved viscoelastic properties and controlling degradation while reducing local inflammation by viscosupplementation and the application of disease-modifying drugs. Furthermore, these treatments can be combined with cell-based therapies to stop disease progression and regenerate the affected cartilage.

The multiple modifications of HA allow the implementation of innovative formulations where the viscoelasticity, degradation, and residence time can be adjusted according to the needs of the patient. Nevertheless, there is ongoing research on other polymers or systems that are better able to imitate, restore, or increase the rheological properties of synovial fluid and that will allow the replacement of HA by more accessible, affordable, and renewable biomaterials. Drug delivery systems have gained more relevance due to their advantages, such as cartilage drug accumulation and extended residence time, which permit increased treatment effects and limit drug toxicity.

Likewise, the search for new scaffolding materials indicated that HA could be replaced by other advanced polymers that are able to mimic and even reinforce the regenerative effects at the joint level. For cartilage regeneration, the optimal scaffolding materials to encapsulate cells are hydrogels, which present high structural and compositional similarity to the cartilage ECM. Hydrogels must preserve the chondrocyte phenotype while promoting cell proliferation and permitting the exchange of nutrients and metabolic wastes. Cellular scaffolding has provided a promising solution to deliver chondrocytes and MSCs into the damaged joint cavity, preventing disease progression and encouraging cartilage regeneration. The viability of this technique relies on the development of engineering hydrogels with high similarity in biological, mechanical, and physicochemical characteristics to those of native cartilage ECM, and that can control cell behavior and fate. However, even if the hydrogel microstructure and mechanical properties can be tuned, and several biochemical factors can be added for MSCs differentiation, chondrogenesis is a process that is difficult to control, due to the complex biochemical and physical cues occurring at different lengths and timescales. To this end, future innovative treatments for OA management may cover all aspects of the disease by simultaneously reducing symptoms and regenerating cartilage.

## Figures and Tables

**Table 1:** Drug delivery systems targeting OA.

Therapeutic Class	Encapsulation Systems	Active Ingredient	Methods/Composition	References
Anti-inflammatory and analgesic drugs	Hydrogels	Loxoprofen	Chitosan/isopropylacrylamide hydrogel	Ahmad [21]
		Dexamethasone	Hyaluronic acid hydrogel	Zhang [22]
		Triamcinolone	Enzymatic activities, responsive TG18 hydrogel (correlate with disease severity)	Joshi [23]
		Prednisolone	Hyaluronic acid/collagen hybrid hydrogel	Mohammadi [24]
		Bupivacaine	Temperature-responsive hydrogel	Kim [25]
		Tramadol	Chitosan nanogel	Barati [26]
		Tramadol	Poloxamer-based binary hydrogel	Dos Santos [27]
		Naproxen, Dexamethason	Gelatin and HA semi-IPN	García-Fernández [30]
	Nanoparticles	Aceclofenac	Solid lipid nanoparticles (SLNs)	Bishnoi [36]
		Celecoxib	Silk fibroin nanoparticles (SFNs)	Crivelli [37]
		Celecoxib	Hyaluronan nanoparticles	El-Gogary [40]
		Dexamethasone	ROS-responsive polythioketal urethane nanoparticles	Zhang [41]
		Celecoxib, dexamethasone and tenoxicam	Terpolymer NPs based on a methacrylic derivative of vitamin E, vinylpyrrolidone and vinylcaprolactam	Pontes-Quero [42]
	Microparticles	Diclofenac Sodium	Lubricating microspheres	Han [57]
		Triamcinolone	Polyester amide microspheres (PEA)	Rudnik-Jansen [50]
		Triamcinolone	PLGA microspheres	Paik [56]
		Celecoxib	Inflammation-responsive polyester amide microspheres (PEA)	Janssen [49]
		Celecoxib	PLGA microspheres with or without cyclodextrin	Cannava [48]
		Etoricoxib	Polycaprolactone microparticles (PCL-MPs)	Arunkumar [47]
		Aceclofenac	PCL or PLGA microspheres	Kaur [46]
		Lornoxicam	Chitosan/tripolyphosphate microspheres (TPP)	Abd-Allah [45]
	Combined systems	Bupivacaine	Microsphere/hydrogel composite (MS/GEL)	Zhang [58]
		Ropivacaine	Nanocapsule/hydrogel composite (NC/GEL)	Khanal [59]
		Methylprednisolone	Microparticle/hydrogel composite (MP/GEL)	Naghizadeh [60]
		Diclofenac	Microsphere/hydrogel composite (MS/GEL)	Qi [61]
	Molybdenum surfaces	Dexamethasone	MbS2 nanosheets	Zhao [62]
	Solid and liquid polymers	Hydrocortisone, triamcinolone y dexamethasone	Cyclodextrins in a solid disk or polymer fluid form	Rivera-Delgado [64]
Antioxidants	Hydrogels	Curcumin	Chitosan/genipin/sodium salts hydrogels	Songkroh [31]
		Quercetin	Polyethylene glycol (PEG)/polyalanine (PA) hydrogel	Mok [32]
		Berberine	pH-responsive chitosan/hyaluronic acid gel	Lu [33]
	Nanoparticles	Curcumin	Terpolymer NPs based on a methacrylic derivative of vitamin E, vinylpyrrolidone and vinyl caprolactam	Pontes-Quero [43]
		Curcumin	Acid-activable poly(β-amino ester) nanoparticles	Kang [44]
		Curcumin	Silk fibroin nanoparticles (SFNs)	Crivelli [37]
		Curcumin	Hyaluronic acid/chitosan nanoparticles	Wang [39]
		Bergenin	Xanthan stabilized silver nanoparticles	Rao [38]
	Microparticles	Curcumin	Silk fibroin/hyaluronic acid microspheres	Sungkhaphan [54]
		Curcumin	Gelatin/silk fibroin microspheres	Ratanavaraporn [53]
		Tetramethylpyrazine	PLGA microspheres	Zhang [52]
		Rutin	Chitosan microspheres	Cosco [51]
	Titanium surfaces	Quercetin	TiO_2_ nanotubes coated with chitosan	Mohan [63]
Immunosuppressive and antirheumatic drugs	Hydrogel	Minocycline	Methoxy polyethylene glycol/caprolactone hydrogel	Park [29]
		Rapamycin	Gelatin hydrogel incorporating drug-micelles	Matsuzaki [28]
	Nanoparticles	Methotrexate	Chitosan nanospheres	Dhanaraj [35]
		Methotrexate	Lipid nanocapsules	Boechat [34]
	Microparticles	Leflunomide	PDLG microspheres	El-Setouhy [55]
	Conjugated systems	Methotrexate	Hyaluronic acid conjugate	Tamura [65]
		Sulfasalazine	Hyaluronic acid conjugate	Kim [66]

**Table 2:** Studies focused on the development of new cell-based therapies for OA treatment.

Type of Cell	Modified Characteristics	System Composition	Relevant Components	Type of Study	Results and Comments	References
Chondrocytes	Without modifications	Chitosan/silk fibroin/eggshell membrane hydrogel	-	In vitro	Biocompatible with chondrocytes	Adali [123]
		Dextran-UPy hydrogel		In vitro and in vivo	Biocompatible with chondrocytes and BMSC	Hou [124]
	Modification of cell environment	Hydrogel with alginate/polyvinyl alcohol semi-IPNs	CS addition	In vitro	Maintenance of chondrocyte phenotype and increased ECM production	Radhakrishnan [121]
		Fibrin/alginate hydrogel	HA and/or CS addition		Increased cell proliferation and ECM production	Little [117]
		Alginate/HA hydrogel	Type II collagen addition		Maintenance of chondrocyte phenotype and increased cell proliferation and ECM production	Mahapatra [119]
		Pullulan/CS hydrogel	Growing CS concentrations		Maintenance of chondrocyte phenotype and increased ECM production	Li [116]
		HA hydrogel	Elastin addition		Increased metabolic activity	Fiorica [113]
		Hydrogel with HA/type II collagen IPNs	TGF-β1 addition		Increased ECM production	Kontturi [115]
		Elastin/HA hydrogel	Growing HA concentrations		Maintenance of chondrocyte phenotype, increased ECM production and reduced degradation	Zhu [122]
		Type I collagen hydrogel	Type I collagen		Transdifferentiation and hypertrophy of chondrocytes	Hu [114]
	Modification of hydrogel physical properties	Alginate/CS hydrogel	Polymer molecular weight		Increased cartilage production, reduced degradation and decrease of inflammation	Ma [118]
Mesenchymal Stem Cells (MSC)	Without modifications	HA hydrogel	-	In vitro and in vivo	MSCs differentiation and chondrocytes and cartilage regeneration	Wu [127]
		PEG/CS hydrogel			MSCs differentiation and chondrocytes and cartilage regeneration	Pascual-Garrido [126]
		Dextran-UPy hydrogel			Simultaneous encapsulation of chondrocytes and BMSCs	Hou [124]
		Chitosan hydrogel			MSCs differentiation and chondrocytes and cartilage regeneration	Jia [125]
	Modification of cell environment	Alginate hydrogel	Adhesion and signaling molecules	In vitro	Increased cell proliferation, chondrogenesis and ECM production	Park [130]
		PEG/HA hydrogel	TGF-β3 and HA addition		Increased chondrogenesis and ECM production	Deng [129]
		PEG hydrogel	Glucosamine addition	In vitro and in vivo	Increased chondrogenesis and ECM production and cartilage regeneration	Yao [131]
		Methacrylated HA hydrogel	CS and type II collagen addition		Increased chondrogenesis and ECM production, reduced degradation and cartilage regeneration	Zhu [132]
		Methacrylated chitosan hydrogel	TGF-β1 and type II colla-gen addition		Increased chondrogenesis and ECM production and cartilage regeneration	Choi [128]
	Modification of hydrogel physical properties	HMW HA hydrogel crosslinked with divinyl sulfone	Crosslinking rate	In vitro	Decreased cell proliferation	Mondal [133]
		PEG/PLA hydrogel	Material stiffness		Decreased cell proliferation	Sun [135]
		Gelatin hydrogel	Material stiffness and degradation	In vitro and in vivo	Increased chondrogenesis, slower hydrogel degradation and cartilage regeneration	Sarem [134]
		Methacrylated collagen hydrogel	Reticular microstructure		Increased chondrogenesis and ECM production and cartilage regeneration	Yang [136]

## Data Availability

Not applicable.
